# Understory upheaval: factors influencing Japanese stiltgrass invasion in forestlands of Tennessee, United States

**DOI:** 10.1186/s40529-018-0236-8

**Published:** 2018-08-06

**Authors:** Lela Z. Culpepper, Hsiao-Hsuan Wang, Tomasz E. Koralewski, William E. Grant, William E. Rogers

**Affiliations:** 1 Department of Ecosystem Science and Management, Texas A&M University, College Station, TX 77843 USA; 2 Department of Wildlife and Fisheries Sciences, Texas A&M University, College Station, TX 77843 USA; 3 Present Address: Department of Wildlife and Fisheries Sciences, Texas A&M University, College Station, TX 77843 USA

**Keywords:** Biological invasion, Boosted regression trees, Forest management, Japanese stiltgrass, *Microstegium vimineum*, Likelihood of invasion

## Abstract

**Background:**

Invasions by non-native plants contribute to loss of ecosystem biodiversity and productivity, modification of biogeochemical cycles, and inhibition of natural regeneration of native species. Japanese stiltgrass (*Microstegium vimineum* (Trin.) A. Campus) is one of the most prevalent invasive grasses in the forestlands of Tennessee, United States. We measured the extent of invasion, identified potential factors affecting invasion, and quantified the relative importance of each factor. We analyzed field data collected by the Forest Inventory and Analysis Program of the U.S. Forest Service to measure the extent of invasion from 2005 to 2011 and identified potential factors affecting invasion during this period using boosted regression trees.

**Results:**

Our results indicated that presence of Japanese stiltgrass on sampled plots increased 50% (from 269 to 404 plots) during the time period. The probability of invasion was correlated with one landscape condition (elevation) (20.5%) and five forest features (including tree species diversity, basal area, stand age, site productivity, and natural regeneration) (79.5%). Boosted regression trees identified the most influential (highly correlated) variables as tree species diversity (30.7%), basal area (22.9%), elevation (20.5%), and stand age (16.7%). Our results suggest that Japanese stiltgrass is likely to continue its invasion in Tennessee forests.

**Conclusions:**

The present model, in addition to correlating the probability of Japanese stiltgrass invasions with current climatic conditions and landscape attributes, could aid in the on-going development of control strategies for confronting Japanese stiltgrass invasions by identifying vulnerable areas that might emerge as a result of likely changes in climatic conditions and land use patterns.

## Background

The presence of invasive plants in forests can cause substantial ecological and economic losses (Ehrenfeld 2003; Mack et al. 2000). Ecologically, invasive plants have decreased native plant diversity, changed community structure, and altered fire regimes (Wang et al. 2016). An economic enterprise that has been adversely affected is timber production in the southeastern U.S. forests. Recent studies suggest invasive species can degrade forest productivity (Wang et al. 2012b). Among invasive plants, grasses are recognized as playing a prominent role in altering native forest communities (D’Antonio and Vitousek 1992).

One such example is the introduction of Japanese stiltgrass (*Microstegium vimineum* (Trin.) A. Campus), which has become a noxious weed in the eastern United States. Originally used as package filling for shipping protection of Chinese porcelain, Japanese stiltgrass is a grass native to southeastern Asia which was first discovered in Tennessee in 1919 (Barden 1987). Like many other invasive exotic species, Japanese stiltgrass exhibits superior competitive ability in novel ecosystems (Mack et al. 2000). In a single season, it can produce thousands of seeds that persist for several years (Gibson et al. 2002). Moist soil conditions are conducive to rapid invasion of Japanese stiltgrass, which often invades roadsides, stream corridors, and trails (Redman 1995). A unique characteristic is its ability to tolerate low-light environments and form monocultures within forest understories (Oswalt et al. 2007). Invasion by Japanese stiltgrass can reduce growth and flowering of native species, suppress native plant communities, alter and suppress insect communities, slow plant succession, and alter nutrient cycling (Emery et al. 2013). Moreover, following disturbance either by a natural (e.g. flooding) or anthropogenic (e.g. timber harvest, mowing) source, Japanese stiltgrass can invade rapidly and replace native plant communities (Nees 2016).

Managing this invasive grass is complicated by its persistent seedbanks in the soil (Gibson et al. 2002) and invaded areas are often too vast for practical, intensive management. Fortunately, although there currently is no biological control agent, mechanical and chemical control methods have advanced in recent years. Judge et al. (2005) experimentally treated Japanese stiltgrass with grass herbicides, originally used for controlling large crabgrass (*Digitaria sanguinalis*), and found the herbicides had a successful kill rate of 87% or greater with repeated application. Flory (2010) noted that hand weeding and two herbicides were effective at reducing re-establishments of Japanese stiltgrass but did little to assist in the recovery of the native species in southern Indiana.

While there is no silver bullet for solving invasive plant problems, statistics and machine learning methods have become invaluable tools for determining the vulnerability of habitats to invasion, identifying underlying causes of invasions, and assisting management efforts (Stohlgren 2002). For example, Cole and Weltzin (2004) found that Japanese stiltgrass presence was only correlated with soil pH, whereas the performance was positively correlated with canopy openness and biomass of other species at Oak Ridge National Environmental Research Park. Moreover, Anderson et al. (2013) suggested that the probability of Japanese stiltgrass presence increased with high human activity, low forest cover, high native species richness, and low basal area of ericaceous shrubs in the southern Blue Ridge Mountains.

In the present study, we analyze an extensive set of field data collected by the U.S. Forest Service to assess the invasion of Japanese stiltgrass within the forestlands of Tennessee, an area which is one of the highest producers of hardwood products in the United States (Young et al. 2007). We first measure the extent of invasion, next identify potential factors affecting invasion, and then quantify the relative importance of each factor.

## Methods

### Study area and data sources

The study area is U.S. State of Tennessee, which is characterized by a temperate to warm climate with mild winter and summer temperatures. Elevations range from 54 to 2025 m, where the highest and lowest points are Clingmans Dome and Mississippi River, respectively. We extracted data on the presence or absence of Japanese stiltgrass collected by the U.S. Forest Service during two field survey cycles (2000–2005 and 2006–2011) using the Southern Nonnative Invasive Plant data Extraction Tool (SNIPET) of the USDA Forest Service (Rudis et al. 2006; USDA 2011). We also collected data on a suite of associated landscape conditions (including elevation, slope, and adjacency to water bodies) and forest features (including stand age, site productivity, tree species diversity, basal area, and natural regeneration), as well as forest management activities (including site preparation, artificial regeneration, cutting, and forestland ownership) and past disturbances (including distance to the nearest road, fire disturbance, animal disturbance, disease disturbance, insect disturbance, human-caused disturbance, and weather disturbance), from the Forest Inventory and Analysis (FIA) program (Bechtold and Patterson 2005). The FIA Program is a forest inventory program in which each state inventory is completed and reported every 5 years in most southeastern states (Bechtold and Patterson 2005). The basic sampling design consists of a lattice of 4047-m^2^ hexagons, with one sample plot located randomly within each hexagon (Bechtold and Patterson 2005; USDA 2011). Each sample plot consists of four subplots of radius 7.32 m which form a cluster consisting of a central subplot and three peripheral subplots equidistant from each other arrayed in a circle of radius 36.58 m centered on the central plot. On each subplot, inventory crews estimate percent cover by target invasive species, and also record a suite of landscape conditions and forest features, as well as past disturbances and forest management activities (Rudis et al. 2006). Thus, the percent cover of invasion in a fixed plot could either increase or decrease.

### Data analysis

We first summarized the data from each of the two surveys by (1) counting the number of sample plots in which Japanese stiltgrass had been detected, (2) noting the percent coverage of Japanese stiltgrass within each of these plots, and (3) mapping the spatial distribution of these plots (using ArcMapTM 10.2, ESRI, Redlands, CA, USA).

We next identified factors potentially influencing the probability of invasion, which included a set of landscape conditions, forest features, forest management activities, and disturbances (Table 1). Landscape conditions included elevation, slope, and adjacency to water bodies within 300 m. Forest features included stand age, site productivity, tree species diversity, basal area, and natural regeneration (growth of existing trees, natural seeding, or both, resulting in a stand at least 50% stocked with live trees of any size). Forest management activities included site preparation (clearing, slash burning, chopping, disking, bedding, or other practices clearly intended to prepare a site for reforestation), artificial regeneration (planting or direct seeding resulting in a stand in at least 50% stocking with trees of any size), and cutting. Disturbance factors include distance between the plot and the nearest road and forest disturbance (such as those caused by animals, disease, fire, insects, weather, and/or humans). We also used the same dataset to compute Shannon’s index of tree species diversity, *Hs*, for each plot (Wang and Grant 2012; Wang et al. 2012a):

$$H_{s} = - \mathop \sum \limits_{i = 1}^{{n_{s} }} \frac{{B_{i} }}{B}ln\left( {\frac{{B_{i} }}{B}} \right)$$

where *B* and *B*_*i*_ are the total stand basal areas and the basal area of trees of species *i*, respectively, and *n*_*s*_ is the number of tree species.

**Table 1:** Descriptions, possible values or units of measure, and means or counts of landscape conditions, forest features, forest management activities, and disturbance factors evaluated as potential factors of site invasion by Japanese stiltgrass in forest plots in Tennessee

Variable	Value or unit of measure	Mean (range) for continuous data/count for categorical data
Landscape conditions		
Elevation	m	213.04 (− 28.04 to 1809.9)
Slope	Degree	10.88 (0.00 to 57.5)
Adjacency to water bodies	No	2138
	Yes	665
Forest features		
Stand age	Years	55.42 (2 to 137)
	L1: 0 to 1.39 m^3^/ha/year	0
	L2: 1.40 to 3.39	256
	L3: 3.50 to 5.94	1840
Site productivity	L4: 5.95 to 8.39	802
	L5: 8.40 to 11.54	290
	L6: 11.55 to 15.74	78
	L7: > 15.74	11
Tree species diversity (Hs)	Shannon’s species diversity	1.88 (0 to 3.02)
Basal area	m^2^/ha	22.53 (0.00 to 332.34)
Natural regeneration^a^	No	3483
	Yes	64
Forest management activities		
Site preparation^a^	No	3508
	Yes	39
Artificial regeneration^a^	No	3389
	Yes	158
Cutting^a^	No	3270
	Yes	277
Forestland ownership	Public	486
	Private	3061
Disturbance factors		
	D3: 92 to 152 m	354
	D4: 153 to 305	578
	D5: 306 to 805	777
Distance to the nearest road	D6: 806 to 1609	333
	D7: 1610 to 4828	116
	D8: 4829 to 8047	11
	D9: > 8047	7
Fire disturbance^a^	No	3511
	Yes	36
Animal disturbance^a^	No	3478
	Yes	69
Disease disturbance^a^	No	3537
	Yes	10
Insect disturbance^a^	No	3421
	Yes	126
Human-caused disturbance^a^	No	3482
	Yes	65
Weather disturbance^a^	No	3470
	Yes	77

^a^Normally within the past 5 years

We associated the data on presence or absence of Japanese stiltgrass (SNIPET) with the data on landscape conditions, forest features, forest management activities, and disturbance factors (FIA Data and Tools) using the FIA plot identification numbers. We then conducted the analysis using boosted regression trees, which combines decision trees and a boosting algorithm with a form of logistic regression (Elith et al. 2008). We fitted the model in R (R Development Core Team 2006 version 2.14.1) using the gbm package version 1.5–7 (Ridgeway 2006). We determined the optimal model following the recommendations of Elith et al. (2008) which was the final model containing at least 1000 trees. We included randomness (with a bag fraction of 0.6) into the models to reduce over-fitting and also to improve accuracy and speed of the model selection process (Friedman 2002). We calculated the response variance explained and the area under the receiver operator characteristic curve (AUC). We evaluated the reliability and validity of the optimal model as fair (0.50 < AUC ≤ 0.75), good (0.75 < AUC ≤ 0.92), very good (0.92 < AUC ≤ 0.97), or excellent (0.97 < AUC ≤ 1.00) based on the value of AUC (Hosmer and Lemeshow 2000). We then used the gbm library to derive the relative influence of each potential explanatory variable in the optimal model and constructed partial dependence plots for the most influential variables (Elith et al. 2008).

## Results

### Historical invasion trends

**Fig. 1:**
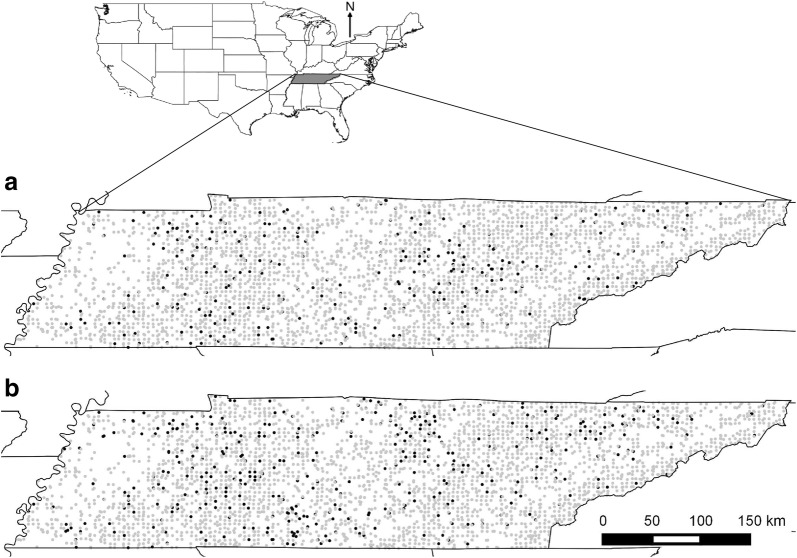
Presence (black dots) and absence (gray dots) of Japanese stiltgrass in forested plots sampled in Tennessee in surveys conducted **a** 2000–2005 and **b** 2006–2011 as part of the Forest Inventory and Analysis Program of the U.S. Forest Service

**Fig. 2:**
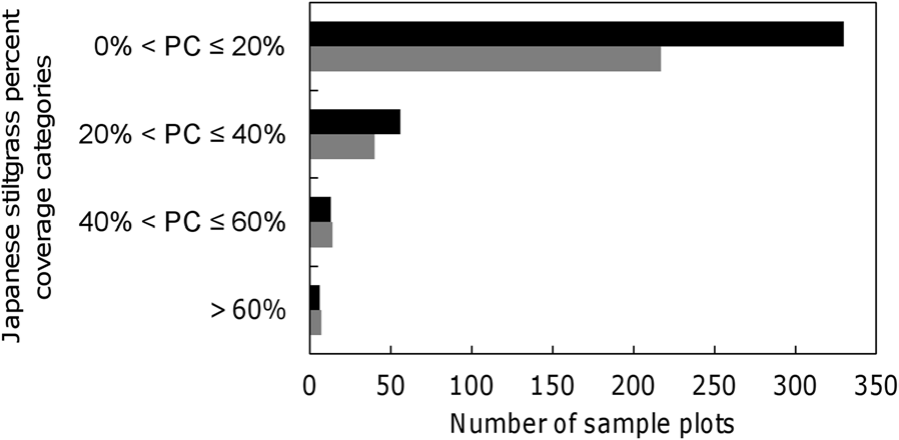
Comparison of the percent coverage (PC) of Japanese stiltgrass during the first survey (2000–2005, gray bar) and the second survey (2006–2011, black bar) conducted by the Forest Inventory and Analysis Program of the U.S. Forest Service. Results are summarized in terms of the number of plots in each of the indicated categories: 0% < PC ≤ 20%, 20% < PC ≤ 40%, 40% < PC ≤ 60%, and PC > 60%

As indicated by the FIA records from 2000 to 2005 and 2006–2011, Japanese stiltgrass spread extensively throughout the forestlands of Tennessee (Fig. 1). The presence of Japanese stiltgrass increased 50% from 269 plots (7.5%) during 2000–2005 to 404 plots (11.3%) during 2006–2011. Although the number of sample plots in the higher percent coverage (PC) categories (40% < PC ≤ 60% and PC > 60%) did not increase, the number of sample plots increased in the lower percent coverage categories (0% < PC ≤ 20% and 20% < PC ≤ 40%) from the first to the second survey (Fig. 2).

### Potential determinants of invasion

We explored 300 combinations of tree complexity (ranging from 5 to 9) and learning rate (ranging from 0.0001 to 0.01), which produced models with between 700 and 1500 trees. The optimal model had a tree complexity of 7, a learning rate of 0.0001, and a total of 1020 trees. Model predictive deviance was 0.809 ± 0.001 with 80.6% of the total response variance explained. The AUC score was 0.773 ± 0.022 (“good” ability to discriminate between species presence and absence). Recursive feature elimination tests showed that twelve variables, including slope, adjacency to water bodies, artificial regeneration, cutting, forestland ownership, distance to the nearest road, fire disturbance, animal disturbance, disease disturbance, insect disturbance, human-caused disturbance, and weather disturbance, could be removed from the model before the resulting predictive deviance exceeded the initial predictive deviance of the model with all variables. Examination of the relative contribution of the predictor variables indicated that the top four accounted for approximately 90% of the contribution in the overall model. Of the four most influential variables, three were forest features (tree species diversity (Hs), basal area, and stand age) and one was a landscape condition (elevation). Species diversity was the most influential variable, contributing 30.7%. Basal area, elevation, and stand age were the second, third, and fourth most important variables, contributing 22.9%, 20.5%, and 16.7%, respectively. Forest features and landscape conditions had total contributions of 79.5% and 20.5%.

**Fig. 3:**
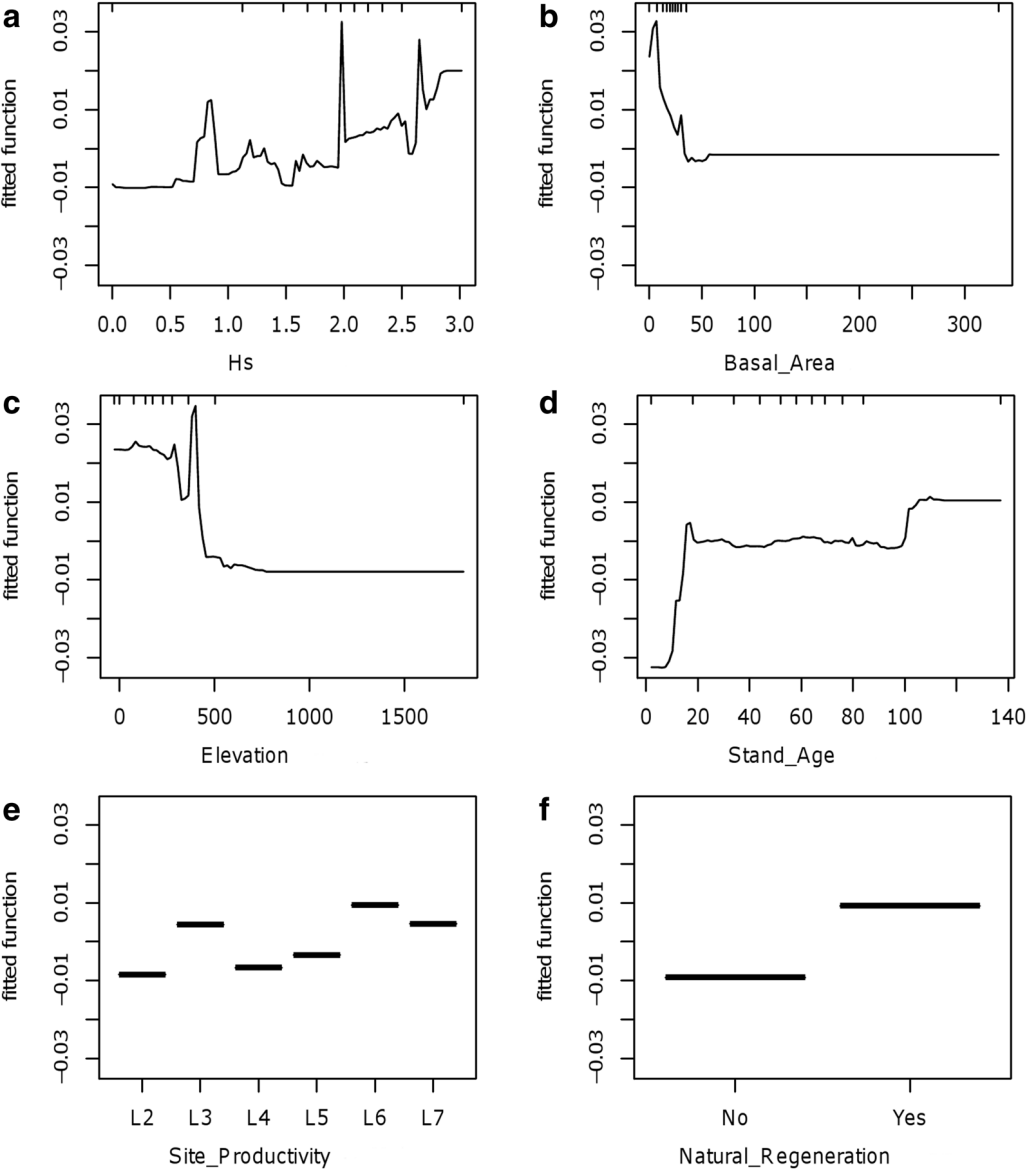
Partial dependence plots for the explanatory variables included in the optimal boosted regression tree model for Japanese stiltgrass presence based on analyses of the six most influential variables. Hash marks at the top of plot **a**–**d** indicate distribution of sample plots along the range of the indicated variable. There is no hash mark at the top of plot **e** and **f** because the partial dependence plots represent categorical variables. X-axes indicate influential variables and their relative contributions (%) in the final model (see Table 1 for the description of variables). Y-axes are based on the logit scale used for the indicated variable

Partial dependence plots indicated that Japanese stiltgrass occurrences were associated with species diversity higher than 0.5 (Fig. 3a). Forest features usually included a basal area per unit area (ha) less than 35 m^2^/ha (Fig. 3b), a stand age either younger than 20 years or older than 100 years (Fig. 3d), site productivity higher than category of L6 (≥ 11.5 m^3^/ha/year) (Fig. 3e), and natural regeneration (Fig. 3f). Occurrences were more likely in plots that were lower than 400 m in elevation (Fig. 3c).

## Discussion

Although Japanese stiltgrass is found throughout most of the eastern United States and in the Caribbean, from New York south to Texas, Florida, Puerto Rico, and the Virgin Islands, it is still spreading into uninvaded areas possessing suitable environmental conditions (Kartesz 1999). Assessing the potential determinants of Japanese stiltgrass invasion will allow managers to design and prioritize their management strategies for specific areas. In particular, our results identify a suite of forest and landscape features that appear to facilitate Japanese stiltgrass invasion thereby facilitating the early detection and eradication of newly established invasions.

While several factors affect site susceptibility to Japanese stiltgrass invasion, higher tree species diversity seems to play the most predominant role. This finding may contradict the common belief that diverse communities are more resistant to invasion (Craig et al. 2015; Forrester 2014; Nord et al. 2010). However, Nord et al. (2010) found that a positive relationship exists between species richness and seedling recruitment of Japanese stiltgrass in central Pennsylvania, suggesting diverse sites are more vulnerable to invasion. Moreover, Anderson et al. (2013) found that high species diversity was conducive to Japanese stiltgrass invasions in the southern Blue Ridge Mountains. Communities with high tree diversity likely provide a high level of resource heterogeneity, including heterogeneous light conditions, thus creating favorable micro-habitats for invasion of shade-tolerant species.

The majority of occurrences of Japanese stiltgrass have been noted on sites with an overstory tree basal area less than 35 m^2^/ha. Knapp et al. (2016) found that light transmittance decreased exponentially while basal area increased in southeast U.S. forests. Thus, lower basal area implies more available light, which coincides with the study of Cole and Weltzin (2004) in eastern Tennessee. These authors concluded that patches of Japanese stiltgrass could occur within the interior forest because of the favorable shift in light conditions.

Apart from suitable forest features, elevation also appears to facilitate Japanese stiltgrass invasions. From our analysis, elevation less than 400 m is associated with its dominance. Existing invasions have occurred in a similar range, between 277 and 1800 m, and suggested that the invasion was associated with low elevation area due to moist to mesic and disturbed soil in East United States (Cole and Weltzin 2004; Craig et al. 2015; Nord et al. 2010).

Forest stands that are either relatively young- or old-aged appear to facilitate Japanese stiltgrass invasions. Several studies (Davies et al. 2007; Kneeshaw and Bergeron 1998; Runkle 1981) have shown that both young and old forests provide structural complexity, large nutrient fluxes, limited competition, and light availability in southeast and eastern U.S. forests. Even though Japanese stiltgrass can tolerate relatively low light and low nutrient fluxes (Gibson et al. 2002), the species accumulate more biomass and nutrients during high-intensity sunflecks (Horton and Neufeld 1998) and in areas with high nutrient fluxes (Cole and Weltzin 2004).

Japanese stiltgrass was more abundant on sites with medium–high site productivity. In general, sites with high productivity provide favorable growing conditions for both native and invasive species (Wang and Grant 2012). Although the Forest Inventory and Analysis (FIA) program defines productivity as the potential of a particular forest stand to grow timber wood and is based on the maximum mean annual increment of fully stocked natural stands (Bechtold and Patterson 2005), productivity reflects soil characteristics and climatic factors (Skovsgaard and Vanclay 2008). Hence, medium–high site productivity appears to favor Japanese stiltgrass invasion.

Our results showed that natural forest regeneration is conducive for the invasion of Japanese stiltgrass. The relationship between seedling establishment and site conditions makes natural regeneration slow and less reliable than artificial regeneration. Shearer and Schmidt (1999) found that even with management treatments that enhanced the rate of natural regeneration, the establishment of the mixed conifer species proceeded slowly for 5 years. This crucial period after a disturbance or the transition between successional stages creates an establishment opportunity for aggressive introduced grass invaders like Japanese stiltgrass. For example, in a highly degraded Hawaiian dry forest, naturally recruited native plants could not out-compete an invasive grass (*Megathyrsus maximus*) without restoration efforts (Ammondt et al. 2013). Thus, in severely disturbed areas, land managers should consider employing artificial regeneration during the initial phases of recovery programs to limit competition between the native and invasive species.

## Conclusions

Even though Japanese stiltgrass continues its range expansion in Tennessee forests, the opportunity exists for reducing the likelihood of invasions via increased monitoring and early control efforts focused on forestlands with high tree species diversity, small basal area, and young stand age, especially at low elevations. Areas with high productivity and natural regeneration also should be targeted for prompt inspection and potential control measures in order to mitigate the ecological impacts of this aggressive understory invader. While there are no direct solutions for invasive plant problems, identifying potential casual factors of invasion can help improve management strategies to limit the spread of non-natives (Wang et al. 2015). In this regard, our model could aid in the on-going development of control strategies for confronting Japanese stiltgrass invasions by identifying vulnerable areas that might emerge as a result of changes in climatic conditions and land use patterns.
